# Advancing osteosarcoma treatment: the impact of intratumoral metabolic reprogramming on immunotherapy

**DOI:** 10.3389/fimmu.2026.1928701

**Published:** 2026-10-07

**Authors:** Jiawei Yin, Shuai Lu, Yikai Liu, Maoqi Gong, Yejun Zha, Xieyuan Jiang

**Affiliations:** 1 Beijing Jishuitan Hospital, Capital Medical University, Beijing, China; 2 Department of Orthopedic Trauma, Beijing Jishuitan Hospital, Capital Medical University, Beijing, China; 3 Beijing Research Institute of Traumatology and Orthopaedics, Beijing, China

**Keywords:** immune checkpoint blockade, immunometabolism, metabolic reprogramming, osteosarcoma, single-cell RNA sequencing, tumor microenvironment

## Abstract

Osteosarcoma (OS) remains difficult to cure once it becomes metastatic, recurrent, or resistant to chemotherapy, and immune checkpoint blockade has provided limited clinical benefit. The metabolic-immune tumor microenvironment (TME) is one barrier to effective immunity because malignant, stromal, and immune cells compete for nutrients and adapt to hypoxia, acidosis, and matrix-related constraints. This review examines how cancer-associated fibroblasts, extracellular matrix, myeloid and lymphoid populations interact with glucose/lactate, glutamine, arginine, and lipid metabolism in OS. Public OS single-cell RNA sequencing datasets are used to illustrate cellular heterogeneity and are interpreted alongside published experimental evidence. OS-specific evidence is most developed for glycolysis and acidosis, glutamine dependence, myeloid regulation, and selected stromal and lipid-related pathways. Arginine-directed approaches, NK-cell immunometabolism, and most metabolism-ICB combinations remain preclinical or are supported mainly by mechanistic evidence from other tumor types. Together, these findings highlight the mechanisms with the strongest current support and the major gaps that remain before clinical translation.

## 1 Introduction

Osteosarcoma (OS) is the most common high-grade primary malignant bone tumor in young patients. Surgery and multi-agent chemotherapy are effective for localized disease, but outcomes remain poor for patients with metastatic, recurrent, or drug-resistant OS (1–5). Unlike many soft-tissue malignancies, OS develops within a mineralized, collagen-rich bone matrix that undergoes continuous remodeling and places distinct spatial constraints on vascular exchange, immune-cell trafficking, and nutrient diffusion. In addition, the strong tendency of OS to metastasize hematogenously to the lungs exposes tumor cells to a second, biologically distinct microenvironment, in which they must adapt to different oxygen levels, immune surveillance, and metabolic conditions. These bone- and lung-specific features may influence metabolic–immune crosstalk and the therapeutic window of metabolism-targeted immunotherapy. Accordingly, this review distinguishes OS-specific findings from mechanistic evidence extrapolated from other tumor types. Although immunotherapy has shown promise in several cancers, immune checkpoint blockade (ICB) has demonstrated limited clinical activity in OS (6, 7). Anti-PD-1 treatment in mouse OS models (8) and a HER2-targeting Listeria vaccine in a canine OS trial (9) provide distinct, model- and modality-specific evidence.

The OS tumor microenvironment (TME) provides a mechanistic basis for understanding resistance to immunotherapy. Malignant osteoblastic cells grow within a matrix-rich, hypoxic, and metabolically stressed bone niche containing cancer-associated fibroblasts (CAFs), extracellular matrix (ECM), endothelial cells, tumor-associated macrophages (TAMs), myeloid-derived suppressor cells (MDSCs), dendritic cells (DCs), T cells, and natural killer (NK) cells (10–12). These cellular and acellular components exchange metabolites, compete for glucose, glutamine, arginine, lipids, and oxygen, and together shape immune signaling and antitumor immune responses.

This review examines how stromal and immune-cell heterogeneity intersects with glucose/lactate, glutamine, arginine, and lipid metabolism in OS. It also uses publicly available single-cell RNA sequencing datasets to illustrate cellular composition and connect those observations with published OS studies. The secondary analysis is not treated as evidence of metabolic flux, causality, or treatment response. Throughout the review, evidence obtained directly in OS is distinguished from mechanisms described mainly in other tumor types, with particular attention to the gaps that still limit clinical translation.

## 2 The osteosarcoma tumor microenvironment as a metabolic-immune ecosystem

The OS TME comprises malignant cells, stromal cells, immune cells, vascular components, ECM, and soluble mediators (10–12). Its clinical significance depends not only on the abundance of these components but also on their functional states. Myeloid enrichment, hypoxia, acidosis, and a dense matrix can limit drug delivery, antigen presentation, and cytotoxic lymphocyte activity, whereas activated DCs, T cells, and NK cells may contribute to tumor control when metabolic constraints are relieved. The major cellular components of the OS TME and their metabolic–immune functions are summarized in **Table 1**.

**Table 1:** Major cellular components of the osteosarcoma tumor microenvironment and their metabolic-immune functions.

Cell type	Key markers	Major functions in OS TME	Metabolic features	Immunological consequences	Therapeutic relevance
CAFs	FAP, LRRC15, TOP2A-positive CAF-related signatures	OS signatures and models support ECM remodeling, stromal drug resistance, and a fibroblast-dependent lung niche (14, 15, 19, 20)	Metabolic states remain incompletely defined; acid and matrix responses are context dependent	Experimental CAF reprogramming increased CD8^+^ T-cell access; CAF functions are heterogeneous	State-specific CAF reprogramming, not indiscriminate depletion; anti-PD-1 signal is OS preclinical (21)
ECM/mesenchymal stromal niche	Collagen I/III, fibronectin, MMPs	OS 3D and pH-responsive models support ECM-associated drug resistance and stromal invasion (19, 92)	Dense matrix and acidity can constrain diffusion and alter stromal signaling	Direct immune-exclusion causality remains untested in OS	ECM or pH modulation is preclinical and requires immune-specific validation
TAMs	CD68, CD163, CD206 and state-specific markers	OS cellular and preclinical evidence supports metastatic, CSF1R, LAT2-CD47, and LIPA-positive programs (29–35)	Glutamine uptake and lipid handling are involved in experimentally supported TAM-related pathways in OS	TAM states can regulate phagocytosis, Tregs, and CD8^+^ T-cell access	CSF1R and LAT2/LIPA pathways are OS preclinical; no established clinical benefit
MDSCs	CD11b, CD33, ARG1; granulocytic and monocytic markers	OS patient-associated and mouse evidence supports IL-18, SDF-1/CXCR4, IL-23, G-CSF, and S100A11-EV pathways (36–40)	ARG, ROS, and NO are mainly non-OS mechanistic context	Suppress T cells and can reduce anti-PD-1 or CAR.GD2 activity in models	Defined cytokine/chemokine targets are OS preclinical; clinical validation is absent
DCs	cDC1: CLEC9A, XCR1, CD141 (human)/CD103 (mouse) cDC2: CD1C, FCER1A, CLEC10A	Direct OS evidence is preclinical; a CD103-positive cDC1 vaccine reduced lung metastases in K7M3 models (72)	DC retention, maturation, and lipid effects are mainly non-OS evidence (70, 71, 99)	Antigen presentation and systemic T-cell priming	cDC1 vaccination plus anti-CTLA-4 has an OS mouse signal, not clinical evidence
T cells/Tregs	CD8, CD4, FOXP3, PD-1, TIM3	OS patient evidence supports prognostic CD8/TIA-1 infiltration and an exhaustion-related phenotype (73, 74)	T-cell use of glucose, glutamine, and arginine is established mainly outside OS	Effector dysfunction and Treg-mediated suppression	Metabolic support plus ICB is hypothesis-generating in OS
NK cells	NKG2D, DNAM-1, TIGIT, CD96, IFN-γ	Human OS samples and murine models support ligand-dependent NK activity, aerosol IL-2, and CD155 blockade (84, 85)	OS-specific metabolic dependencies remain undefined	Model-specific cytotoxicity and cytokine production	NK-cell strategies are OS preclinical and context dependent

Evidence from other tumor types is discussed only as mechanistic context unless it has been validated in OS, because differences in cellular origin, bone-niche biology, age distribution, and metastatic patterns may affect the therapeutic window of immunometabolic interventions.

### 2.1 CAFs, ECM, and the matrix-rich OS niche

CAFs contribute to tumor progression through extracellular matrix (ECM) remodeling, angiogenesis, immune regulation, and metabolic support (13). In OS, CAF-related signatures have been associated with immune suppression and adverse clinical features (14, 15). The dense collagen- and mineral-rich OS matrix may also influence vascular exchange and drug penetration, although these effects have not been systematically quantified in patients. Evidence from other tumors shows that CAF-derived ECM can limit T-cell recruitment and activation, but the magnitude of this effect in OS depends on the stromal state and model (16–18).

Several OS-specific studies have directly examined these stromal effects. In a three-dimensional co-culture model, type I and III collagen and fibronectin accumulated at the interface between OS cells and mesenchymal stromal cells and reduced doxorubicin cytotoxicity (19). Single-cell and mechanistic analyses further identified a CXCL14-integrin α11β1 axis through which OS cells activated fibroblast contractility, matrix remodeling, and a supportive lung metastatic niche. Blocking this axis increased CD8^+^ T-cell activity and reduced lung metastasis in experimental models (20). In an orthotopic OS model, a sequential hydrogel that reprogrammed CAFs increased T-cell infiltration and enhanced the activity of anti-PD-1 treatment (21).

CAF function is not uniformly immunosuppressive. Fibroblast states differ in origin and function, and some may promote immune-cell recruitment, support antigen presentation, or restrain tumor growth (22–25). Their cellular state, anatomical site, and treatment context should therefore be considered when evaluating stromal depletion or reprogramming in OS (**Figure 1**).

**Figure 1:**
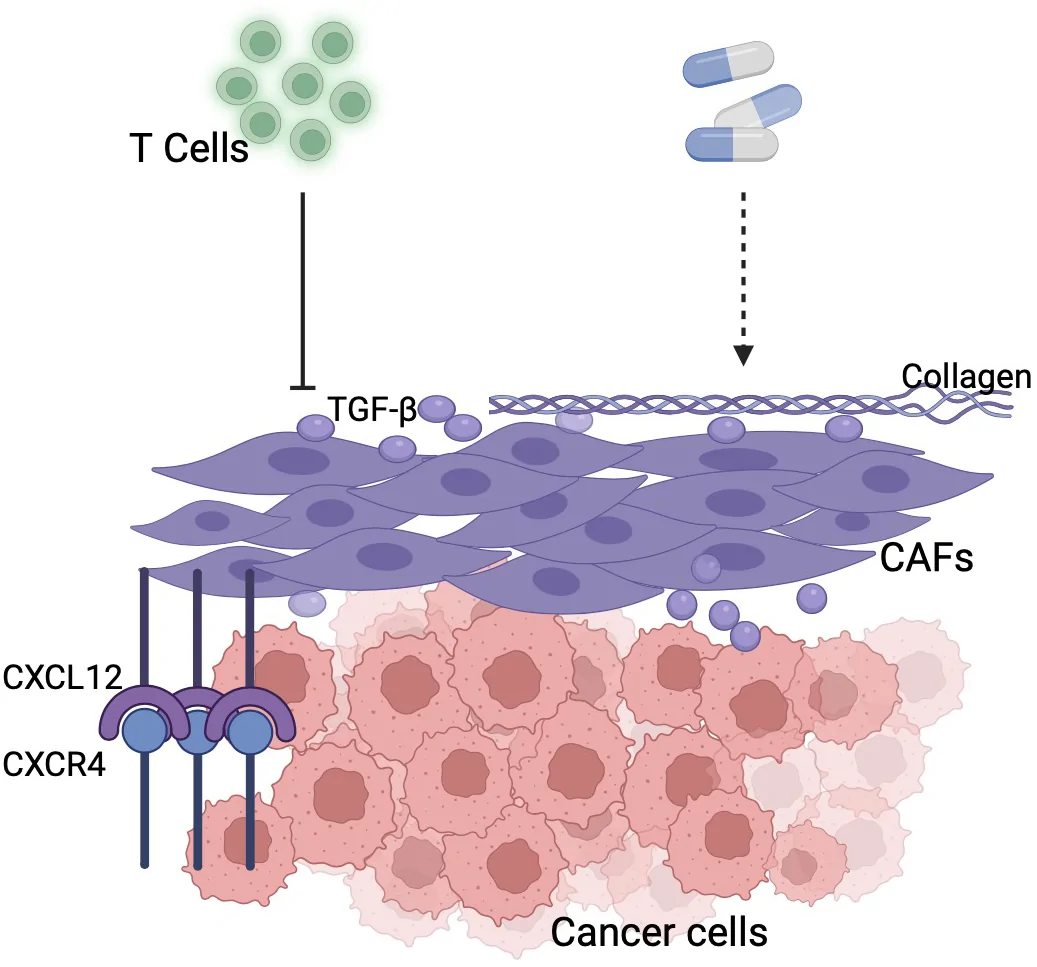
Functions of cancer-associated fibroblasts (CAFs) in the tumor microenvironment (TME). CAFs contribute to tumor progression through ligand-receptor signaling with tumor cells, extracellular matrix (ECM) remodeling, suppression or depletion of cytotoxic T lymphocytes (CTLs), and promotion of drug resistance. In osteosarcoma, these functions should be interpreted together with the bone matrix-rich niche and chemotherapy-resistant ECM deposition.

### 2.2 Myeloid populations: TAMs, MDSCs, and dendritic cells

Myeloid cells are major regulators of the OS immune microenvironment. Tumor-associated macrophages (TAMs) occupy a range of inflammatory, reparative, and immunosuppressive states that are not adequately represented by a binary M1/M2 classification. Across cancers, TAM states have been linked to therapy resistance, exosome-driven immunosuppression, and clinical outcome (26–28). These findings include non-OS evidence; the roles of TAMs in OS remain context dependent (29). Macrophage-derived CCL18 promotes OS proliferation and metastasis through the EP300/UCA1/Wnt/β-catenin axis, whereas COX-2/STAT3 signaling and M2-like TAM exosomes support lung metastasis and invasive behavior (30–32). The crosstalk between M2-like TAMs and OS cells is illustrated in **Figure 2**.

**Figure 2:**
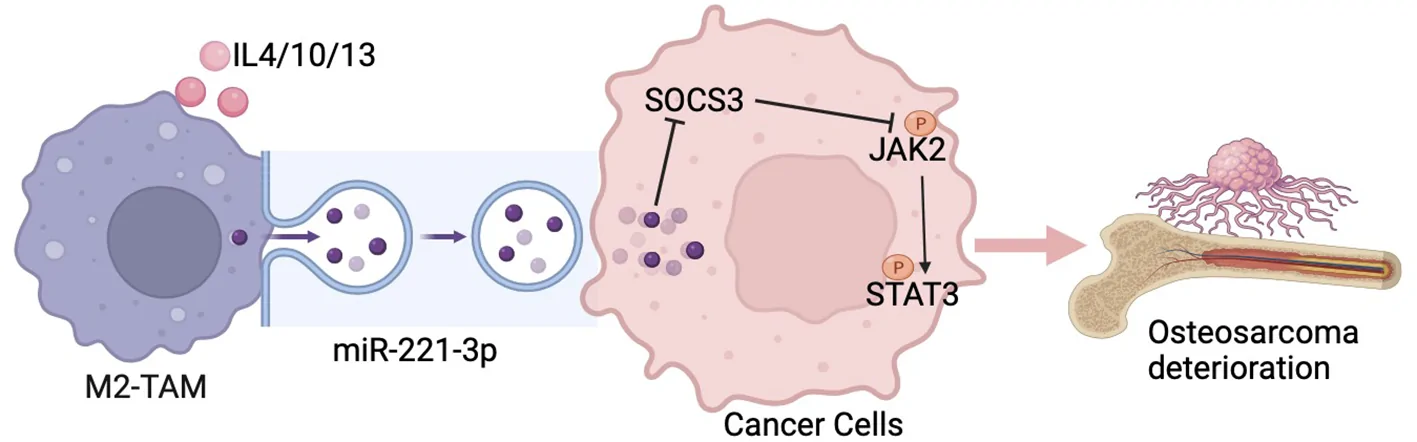
Crosstalk between M2-polarized tumor-associated macrophages (M2-TAMs) and osteosarcoma (OS) cells. OS cells can release factors that favor macrophage polarization toward an M2-like phenotype. M2-TAM-derived exosomes and cytokines may in turn promote OS cell proliferation, invasion, migration, and metastatic progression, supporting a myeloid-enriched immunosuppressive niche. This schematic illustrates an M2-like TAM pathway reported in OS and does not encompass the full spectrum of macrophage states in the tumor microenvironment.

Recent OS studies have identified several macrophage pathways that influence tumor growth and immune regulation. In an orthotopic LM8 model, the CSF1R inhibitor pexidartinib altered the TAM compartment, reduced FOXP3^+^ Tregs, increased CD8^+^ T-cell infiltration, and suppressed primary growth and lung metastasis (33). After chemotherapy, macrophage-derived IL-18 increased leucine and glutamine uptake in OS cells through LAT2, an amino acid transporter. This activated mTORC1-c-Myc signaling and increased CD47 expression. LAT2 inhibition increased macrophage infiltration and phagocytosis and sensitized OS to doxorubicin in mice (34). A separate study identified LIPA-positive macrophages in human, mouse, and patient-derived xenograft OS samples and linked this state to T-cell suppression in functional models (35).

Direct OS evidence also supports a role for myeloid-derived suppressor cells (MDSCs). MDSCs were elevated in blood from patients with OS, while experiments in tumor-bearing mice linked their accumulation to IL-18 and showed that IL-18 blockade enhanced anti-PD-1 activity (36). OS tissues contained CXCR4-positive MDSCs, and CXCR4 antagonism improved anti-PD-1 responses in a murine model (37). Genetic and pharmacological studies identified a GRM4-regulated IL-23/IL-12 program in infiltrating myeloid cells (38). Tumor-derived G-CSF expanded suppressive MDSCs and reduced the durability of CAR.GD2 T cells in an orthotopic OS model (39). OS extracellular vesicles containing S100A11 also promoted a lung premetastatic niche by recruiting granulocytic MDSCs through macrophage-dependent CXCL2 signaling (40).

MDSC nomenclature and characterization frameworks derive from broader myeloid biology (41–46). Non-OS studies link myeloid development to IRF8 signaling (47, 48), suppressive states to PERK-STING regulation (49), and myeloid mobilization to angiogenesis and G-CSF (50, 51). Reactive nitrogen species can disrupt antigen recognition and lymphocyte access (52–55), while arginine depletion, lactate, and hypoxia provide metabolic routes to myeloid-mediated immune suppression (56–59). These mechanisms provide context for OS, but they should not be described as OS-specific without direct validation. OS studies therefore support specific cytokine, chemokine, and metastatic niche mechanisms involving MDSCs, although their metabolic regulation in OS remains incompletely understood.

Dendritic cells (DCs) link innate and adaptive immunity through antigen presentation and T-cell priming (60–69). Non-OS studies have reported unproductive T-cell retention in tumor-DC networks and impaired maturation of tumor-infiltrating DCs (70, 71). Direct OS evidence is mainly preclinical. In K7M3 models, a tumor-lysate-loaded CD103-positive conventional type 1 DC vaccine generated systemic T-cell responses, reduced established lung metastases, and showed greater activity when combined with anti-CTLA-4 (72). Clinical efficacy and predictive DC biomarkers have not been established in OS.

### 2.3 Lymphoid populations: T cells and NK cells under metabolic stress

OS patient data show that lymphocyte abundance and functional state are clinically relevant. In a cohort of 129 patients with localized OS, CD8/TIA-1 infiltration was independently associated with survival, whereas PD-L1 expression in immune cells identified a poorer-outcome subgroup among CD8-positive tumors (73). A separate study of patient-derived tumor-infiltrating lymphocytes found increased PD-1 and TIM3 expression and impaired CD8^+^ T-cell function. TIM3 blockade restored T-cell alloreactivity ex vivo and reduced tumor growth in a xenograft model (74).

The metabolic requirements of these lymphocytes are better established outside OS. Glycolysis, glutamine uptake, and arginine availability support T-cell activation and effector function, whereas regulatory T cells (Tregs) can remain active under different metabolic conditions (75–82). These studies suggest that nonselective metabolic inhibition may weaken antitumor immunity, but they do not show that nutrient competition directly causes the exhaustion phenotype observed in OS. In B16 melanoma models, local IL-21 enhanced effector T-cell activity and reduced Treg accumulation, providing a non-OS example of cytokine-mediated lymphoid regulation (83).

OS-specific evidence for natural killer (NK) cells includes human tissue studies and preclinical treatment models. OS cell lines and 103 patient samples expressed variable levels of NKG2D ligands, and aerosol IL-2 increased pulmonary NK-cell accumulation and improved NK-cell activity in a lung metastasis model (84). In a murine model of OS relapse after allogeneic bone marrow transplantation, CD155 blockade enhanced allogeneic NK-cell cytotoxicity through DNAM-1 and improved survival (85).

General NK-cell studies describe cytokine production, target recognition, and susceptibility to suppressive signaling (86–90). However, the effects of glucose, lactate, amino-acid availability, and lipid stress on NK cells have not been directly resolved in OS. Whether metabolic interventions can improve NK-cell therapy in OS remains unknown and requires direct testing (**Figure 3**).

**Figure 3:**
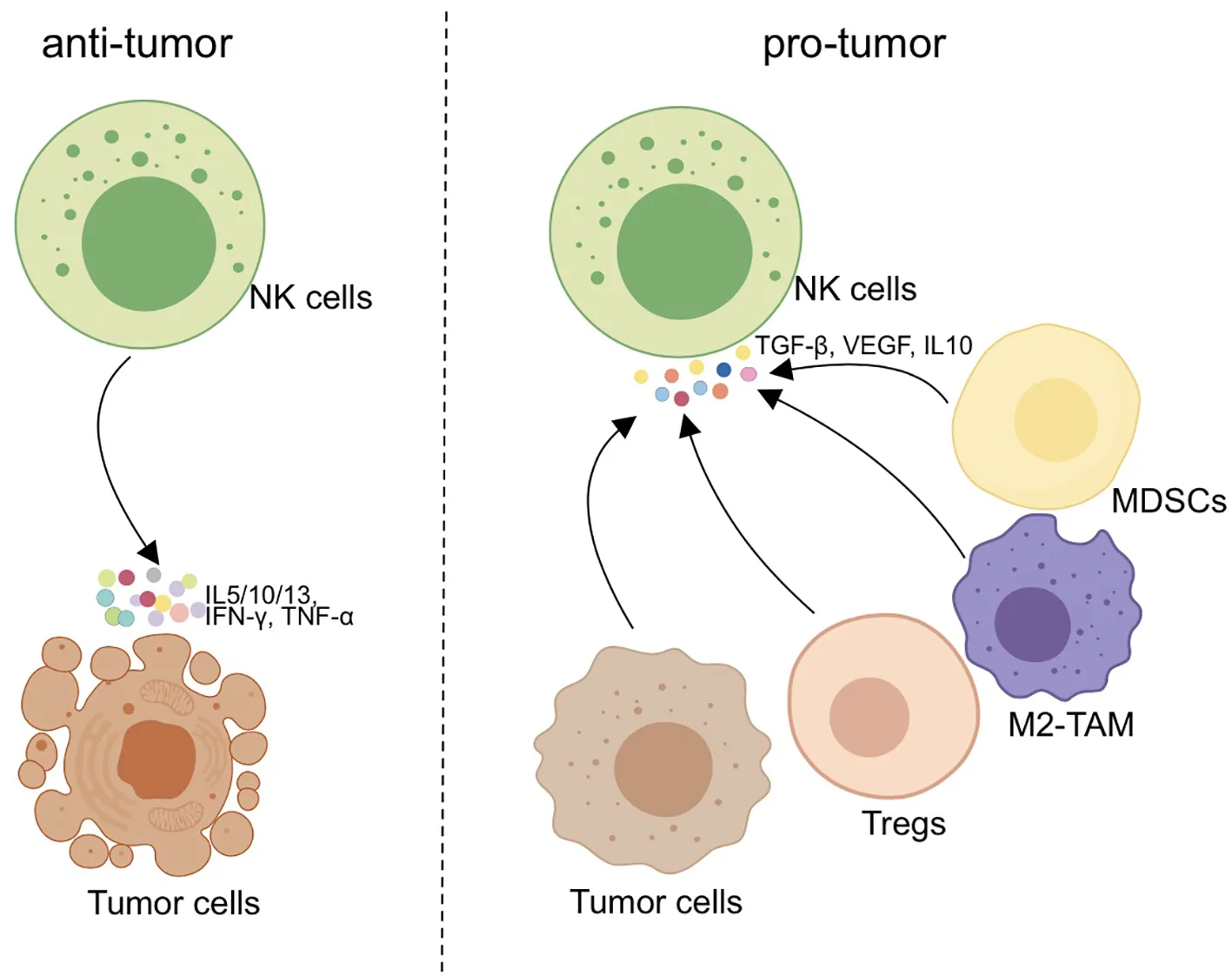
Context-dependent roles of natural killer (NK) cells in the tumor microenvironment. NK cells exert antitumor activity through cytokine production and cytotoxic killing. However, tumor cells, stromal cells, and suppressive immune populations can release inhibitory signals that reduce NK-cell activation and cytotoxic function. The extent to which these mechanisms dominate in OS requires further validation.

### 2.4 Hypoxia, acidosis, and immune exclusion

Hypoxia and acidosis have direct effects on OS tumor and stromal cells. In OS cells, hypoxia-induced HIF-1α increased the expression of NDUFA4L2, a HIF-1α-regulated factor associated with reduced ROS production, thereby promoting migration, invasion, proliferation, and epithelial-mesenchymal transition in cellular assays (91). These findings support a tumor-cell adaptation mechanism but do not establish an immune effect.

Acidosis has also been examined in patient samples and OS models. At diagnosis, serum IL-6 correlated with 18F-FDG uptake and disease-free survival. Subcutaneous and orthotopic xenografts showed an acidic microenvironment, and three-dimensional microfluidic experiments linked acid-stimulated mesenchymal stromal cell IL-6 signaling to OS invasion and metastatic behavior (92). Acid-adapted OS cells also accumulated lipid droplets and showed increased sphingosine 1-phosphate signaling. FTY720 impaired acid-induced survival and migration *in vitro* and, when combined with a low-serine/glycine diet, reduced tumor growth in xenograft models (93).

Evidence from non-OS tumor models suggests that hypoxia, lactate accumulation, and low extracellular pH can alter macrophage states and inhibit effector lymphocytes (57, 58, 94–96). In non-OS models, VISTA binding to PSGL-1 is enhanced under acidic conditions (97), while VISTA and PD-1 can independently regulate T-cell responses (98). Neither mechanism has been directly established in OS. The immune consequences of acidosis should therefore be separated from the better-supported tumor and stromal effects in OS.

Regional oxygen and metabolite gradients are plausible within the mineralized and vascularly abnormal OS niche, but their spatial distribution has not been comprehensively mapped in patients. Strategies that buffer acidity, limit lactate transport, or modify stromal and vascular barriers remain experimental. Their effects on defined OS immune-cell populations and responses to ICB have not yet been established.

## 3 Single-cell landscape of osteosarcoma

To provide cellular context for the metabolic and immune interactions discussed in this review, we performed a secondary analysis of two publicly available OS single-cell RNA-sequencing datasets. The aim was to visualize the major cell populations in the OS microenvironment (**Figure 4A**) and examine differences in cellular composition across samples (**Figure 5**). GSE152048 included 100,987 cells from seven primary, two recurrent, and two lung metastatic OS lesions (100). GSE162454 included 29,278 cells from six treatment-naive primary OS samples and incorporated multiplex immunofluorescence validation of selected cell subsets (101). This analysis is descriptive and hypothesis-generating and is not intended to establish metabolic flux, treatment response, or causal mechanisms.

**Figure 4:**
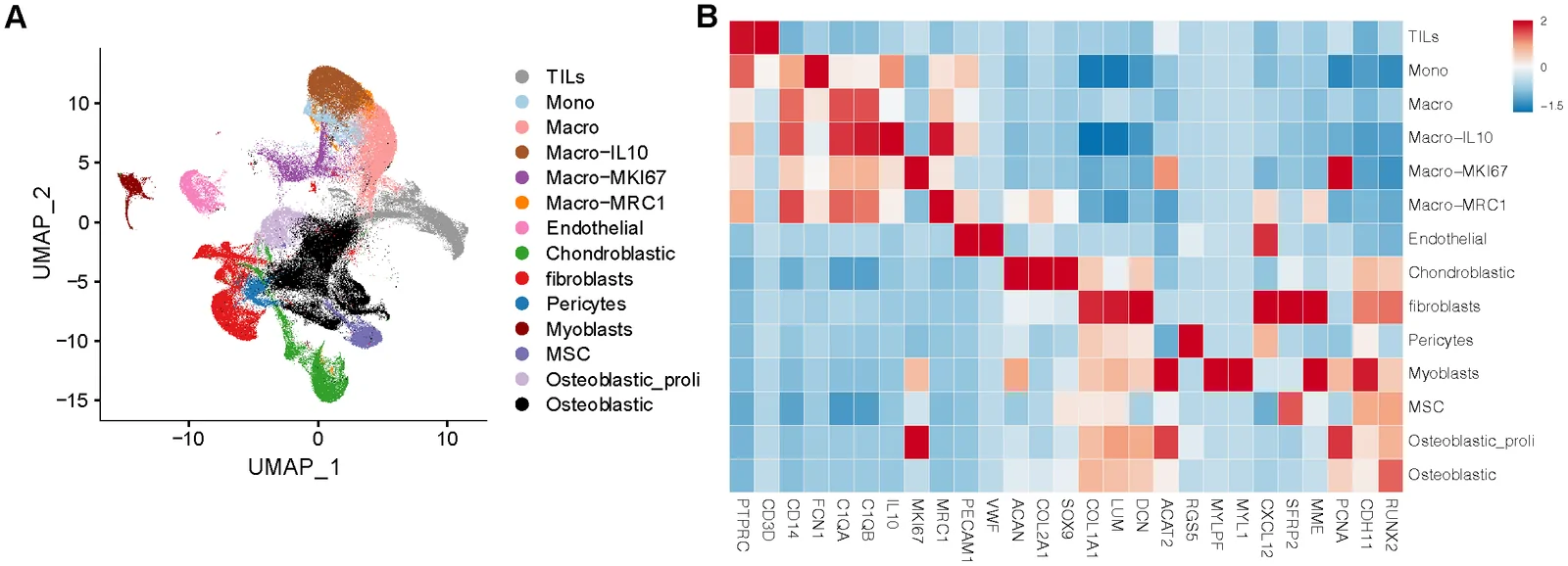
Single-cell landscape of the osteosarcoma tumor microenvironment. **(A)** Uniform manifold approximation and projection (UMAP) visualization showing major annotated OS TME cell populations identified from public single-cell RNA sequencing datasets GSE162454 and GSE152048 (100, 101). **(B)** Heatmap showing marker-gene expression across annotated cell populations. These visualizations represent a secondary reanalysis of publicly available datasets and provide a hypothesis-generating framework for illustrating cellular heterogeneity in OS. TILs, tumor-infiltrating lymphocytes; Mono, monocytes; Macro, macrophages; Macro-IL10, IL10-expressing macrophages; Macro-MKI67, proliferating MKI67-expressing macrophages; Macro-MRC1, MRC1-expressing macrophages; MSC, mesenchymal stromal cells; Osteoblastic_proli, proliferating osteoblastic cells.

**Figure 5:**
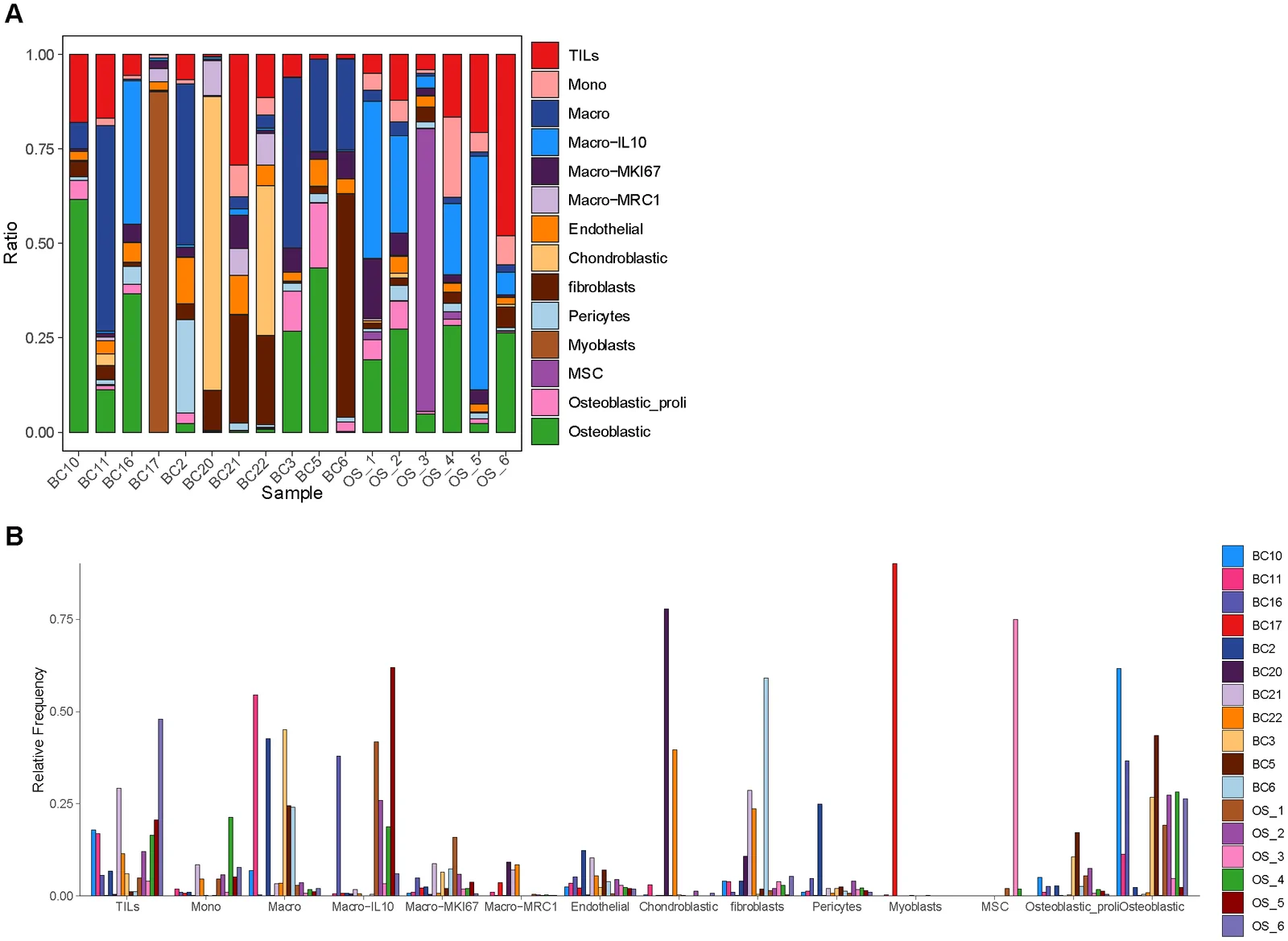
Cellular composition of osteosarcoma samples based on single-cell analysis. **(A)** Stacked bar plots showing the relative proportions of the annotated cell populations within individual osteosarcoma samples from GSE162454 and GSE152048 (100, 101). **(B)** Bar plots showing the distribution of individual cell populations across samples. These visualizations represent a secondary reanalysis of publicly available datasets and are intended to illustrate inter-sample cellular heterogeneity rather than establish causal metabolic mechanisms. TILs, tumor-infiltrating lymphocytes; Mono, monocytes; Macro, macrophages; Macro-IL10, IL10-expressing macrophages; Macro-MKI67, proliferating MKI67-expressing macrophages; Macro-MRC1, MRC1-expressing macrophages; MSC, mesenchymal stromal cells; Osteoblastic_proli, proliferating osteoblastic cells.

A Seurat-based workflow was used for the single-cell RNA-sequencing analysis. Quality control was based on the number of detected genes, unique molecular identifier counts, and the proportion of mitochondrial transcripts. Following normalization, dimensionality reduction, unsupervised clustering, marker-based cell annotation, and uniform manifold approximation and projection were performed to visualize the major cell populations.

Cell populations were annotated using representative marker-gene expression patterns (**Figure 4B**). Representative markers included PTPRC and CD3D for tumor-infiltrating lymphocytes; CD14 and FCN1 for monocytes; C1QA and C1QB for macrophages; IL10, MKI67, and MRC1 for the corresponding macrophage subpopulations; PECAM1 and VWF for endothelial cells; ACAN, COL2A1, and SOX9 for chondroblastic cells; COL1A1, LUM, and DCN for fibroblasts; ACTA2 and RGS5 for pericytes; MYLPF and MYL1 for myoblasts; CXCL12, SFRP2, and MME for mesenchymal stromal cells; and PCNA, CDH11, and RUNX2 for proliferating and non-proliferating osteoblastic populations. Cell annotation was based on the combined expression patterns of multiple markers rather than on any single gene.

Independent single-cell and spatial work has since mapped chemotherapy-associated cell states and a spatial niche at the front of necrotic regions in human OS (102). These studies support cellular and spatial heterogeneity, but transcript abundance alone does not quantify pathway flux or therapeutic dependence. Detailed parameters that were not retained during the original analysis were not reconstructed or inferred.

Published OS evidence links myeloid heterogeneity to several experimentally defined processes, including macrophage-driven LAT2-CD47 regulation, cytokine-dependent MDSC accumulation, and LIPA-positive TAM states (34–37). The present secondary reanalysis therefore provides cellular context for these published findings but does not establish their metabolic activity or causal role.

Published OS studies also connect stromal heterogeneity with ECM deposition, fibroblast-mediated matrix remodeling, and acid-stimulated mesenchymal stromal responses (19, 20, 92). The secondary single-cell analysis identified fibroblast and mesenchymal stromal cell clusters that provide cellular context for these mechanisms, but do not establish pathway activity (**Figures 4**, **5**).

For lymphoid populations, OS patient studies support CD8^+^ T-cell infiltration and exhaustion-related phenotypes, while NK-cell studies provide model-specific evidence of antitumor activity (73, 74, 84, 85). The observed variation between samples does not indicate which metabolic intervention, if any, would benefit lymphoid function in an individual tumor.

## 4 Metabolic crosstalk in the osteosarcoma TME

Metabolic crosstalk in OS can be organized into four interacting axes: glucose/lactate, glutamine, arginine, and lipid metabolism. For each axis, the following sections separate direct OS findings from non-OS mechanisms and identify whether therapeutic evidence is cellular, preclinical, or clinical. The corresponding metabolic pathways, OS-specific evidence, and potential therapeutic strategies are summarized in **Table 2**.

**Table 2:** Metabolic pathways implicated in osteosarcoma progression and immune regulation.

Metabolic pathway	Key molecules	Evidence in osteosarcoma	Effect on immune microenvironment	Potential therapeutic strategy
Glucose/lactate	LDHA, HCAR1, MCT1, MCT4, HIF-1α	OS cellular/preclinical and limited patient-associated evidence supports glycolytic, lactate, and acidosis programs (92, 107–110)	Direct OS tumor/stromal effects; T/NK and TAM mechanisms are mainly non-OS (57, 96, 111, 112)	HCAR1 or pH-directed strategies are OS preclinical; MCT-ICB combinations require OS validation
Glutamine	GLS1, LAT2, SLC38A5	OS tissue and preclinical evidence supports GLS1, LAT2-CD47, and SLC38A5 pathways (34, 122–124)	OS evidence links macrophage-derived IL-18 to LAT2-CD47 signaling; broader macrophage and T-cell metabolic effects are mainly supported by non-OS studies	GLS1 inhibition is OS preclinical; ICB combinations remain non-OS
Arginine	ARG1/2, NOS, SLC7A3, SIRPA	OS preclinical evidence supports L-arginine plus anti-PD-L1 and SIRPA-SP1-SLC7A3 signaling (132, 133)	OS myeloid ARG1 evidence remains limited	Cell- and context-specific intervention; no human clinical evidence
Lipid metabolism	FASN, FABP5, SQLE, S1P, LIPA	OS cellular, patient-associated, and preclinical evidence supports tumor and TAM lipid programs (35, 93, 145–147)	Direct T-cell and DC evidence is largely non-OS	Target selection must distinguish tumor-cell, TAM, and lymphocyte requirements
Hypoxia/acidosis	HIF-1α, NDUFA4L2, IL-6, VISTA	OS cellular and pH-imaged preclinical evidence supports HIF-1α adaptation and acid-stromal signaling (91, 92)	Low-pH immune suppression and VISTA-related T-cell regulation are supported mainly by non-OS evidence (57, 58, 94–98)	pH or stromal modulation is preclinical; OS immune effects remain unverified

### 4.1 Glucose metabolism, lactate accumulation, and acidosis

Aerobic glycolysis is a general feature of many cancers (94, 103–106). Direct OS studies have linked circ-CTNNB1/RBM15 signaling and YTHDF3-dependent stabilization of PGK1 with glycolysis and aggressive cellular behavior (107, 108). Targeted metabolomic analyses further showed that nutrient dependence changes across OS progression models. Metastatic cells showed faster metabolic flux, whereas malignant non-metastatic OS cells were relatively resistant to glycolytic inhibition and showed a stronger dependence on glutamine (109).

OS evidence also links glycolysis and acidosis to stromal biology. In patients, serum IL-6 correlated with 18F-FDG uptake, an indirect measure of tumor glycolysis, and with disease-free survival. In pH-imaged xenografts and microfluidic models, acidosis activated NF-κB-related IL-6 production in mesenchymal stromal cells and increased OS invasiveness (92). Acid-adapted OS cells also accumulated lipid droplets and activated sphingosine 1-phosphate signaling, linking pH stress to lipid survival programs (93).

Direct OS evidence for lactate signaling is emerging. Lactate-activated HCAR1 (hydroxycarboxylic acid receptor 1), a lactate-responsive receptor, promoted OS progression through a β-arrestin2-PP2A-STAT1/2 pathway, and PP2A inhibition reduced aggressive cellular phenotypes and showed synergy with cisplatin in preclinical experiments (110). By contrast, evidence that lactate polarizes TAMs or directly suppresses T and NK cells is derived mainly from non-OS tumor models (57, 96, 111, 112).

LDH and monocarboxylate transporters are potential therapeutic targets, but their effects are cell-type dependent. LDH activity supports glycolytic tumor cells and also contributes to T-cell differentiation and function (112–114). MCT4 inhibition increased CD8^+^ T-cell infiltration and enhanced anti-PD-1 activity in hepatocellular carcinoma, not OS (115). The antitumor and immune effects of MCT inhibition have not been established in OS.

Current OS studies support a role for glycolytic and lactate-dependent tumor programs, together with stromal responses to acidosis. Therapeutic strategies targeting HCAR1 or sphingosine 1-phosphate remain preclinical, while combinations of LDH or MCT inhibition with immunotherapy still require validation in OS.

### 4.2 Glutamine metabolism

Glutamine supplies carbon and nitrogen for biosynthesis, redox control, nucleotide synthesis, and mitochondrial function (116–120). In MYC-dependent mouse osteogenic sarcoma cells, glutamine stimulated mitochondrial respiration through aminotransferase-dependent metabolism; inhibiting aminotransferases reduced proliferation and survival (121). Preclinical studies showed reduced tumor growth and metastatic progression after GLS1 inhibition (122), while OS tissue studies have documented GLS1 expression (123). Progression-dependent metabolic profiling also showed that OS models differ in their relative dependence on glycolysis and glutamine (109).

Glutamine metabolism can also regulate immune evasion in OS. Chemotherapy-induced macrophage IL-18 increased LAT2-mediated leucine and glutamine uptake in OS cells, activating mTORC1-c-Myc signaling and CD47 expression (34). The amino acid transporter SLC38A5 was upregulated in OS cohorts and promoted glutamine-dependent PI3K-AKT-mTOR/SREBP1-SCD1 signaling, ferroptosis resistance, and xenograft growth (124).

Direct preclinical evidence is available for GLS1 inhibition in OS. CB-839 reduced OS growth and metastatic progression, and its combination with metformin altered glycolysis and tricarboxylic acid cycle activity (122). LAT2 and SLC38A5 are additional transporters involved in OS amino acid metabolism, although neither has been clinically validated as a therapeutic target.

Immune effects require separate consideration. Studies outside OS show that glutamine metabolism influences macrophage polarization, suppressive myeloid cells, and T-cell adaptation (76–79, 81, 125–128). Glutamine deprivation induced PD-L1 expression in renal cancer cells (129), while glutamine blockade enhanced ICB in other non-OS models (81, 130). Whether a comparable therapeutic window exists in OS remains unknown.

### 4.3 Arginine metabolism

Arginine availability supports CD8^+^ T-cell survival and antitumor activity (82). In other tumor settings, ARG-expressing MDSCs and TAMs can deplete extracellular arginine and impair T-cell responses (58, 59). In non-OS tumor models, lactate can induce ARG1 expression in tumor-associated macrophages (57), while spatial metabolic organization may further influence nutrient availability across tumor regions (131). Whether these processes interact in OS remains unclear.

OS studies illustrate the context-dependent role of arginine in both immune and tumor cells. L-arginine supplementation combined with anti-PD-L1 increased CD8^+^ T-cell abundance and function and prolonged survival in immunocompetent orthotopic and metastatic OS models (132). In OS tissues and experimental systems, SIRPA stabilized SP1 and increased SLC7A3-dependent arginine uptake, thereby promoting migration and metastasis. Combined SIRPA blockade and arginase reduced metastasis in xenograft models (133).

These results do not define a preferred clinical strategy. Arginine deprivation can suppress auxotrophic tumor cells but may also impair T-cell function or select for compensatory synthesis (134, 135). Myeloid arginase inhibition has immunological support in non-OS models (136). Arginine-targeted approaches in OS will therefore need to account for the different requirements of tumor and immune cells. Human efficacy remains unknown, and the specific role of myeloid ARG1 in OS has not yet been established.

### 4.4 Lipid metabolism and immune dysfunction

Lipid metabolism supports membrane synthesis, redox control, signaling, and therapy resistance across cancers (137–144). In OS cells, circREOS restrained HuR-mediated MYC activation, reduced FASN expression and lipid accumulation, and suppressed proliferation and invasion (145). NSUN2 also promoted fatty acid metabolism and OS progression by stabilizing FABP5 mRNA through m^5^C modification (146). A multi-omics and single-cell study identified squalene epoxidase (SQLE) as a high-risk, chemoresistance-associated OS vulnerability. SQLE suppression or pharmacological inhibition reduced malignant behavior and improved chemosensitivity in preclinical models (147).

In OS models, acidosis also alters lipid metabolism within the tumor microenvironment. Acidosis increased lipid-droplet accumulation and sphingosine 1-phosphate signaling in OS models, while FTY720-based intervention reduced acid-adapted survival and xenograft growth (93). LIPA-positive TAMs identified across human, mouse, and patient-derived xenograft samples promoted tumor progression and T-cell suppression in functional experiments (35).

Most evidence linking other lipid pathways to immune-cell function comes from non-OS tumor models. Lipid loading can impair DC antigen presentation, and cholesterol or oxidized lipid uptake can contribute to CD8^+^ T-cell dysfunction (99, 148–151). CD36-dependent Treg survival and fatty-acid oxidation in memory or tissue-resident T cells further show that lipid targeting can have opposing effects across immune subsets (152–157).

Current OS studies support roles for FASN, FABP5, SQLE, sphingosine 1-phosphate signaling, and LIPA-positive TAMs in tumor metabolism or immune regulation. In contrast, CD36, cPLA2α, fatty acid oxidation, and their potential combinations with ICB have been studied mainly in other tumor types and still require direct evaluation in OS.

## 5 Therapeutic implications: targeting metabolism to improve immunotherapy

OS-specific studies provide support for selected targets involving glycolysis and acidosis, glutamine metabolism, myeloid pathways, arginine availability, and lipid metabolism, although the therapeutic evidence remains predominantly preclinical (**Figure 6**). Most evidence for metabolism-ICB combinations still comes from non-OS models, and these approaches should not be presented as established treatments for OS. Representative metabolism-targeted strategies and their potential combinations with immunotherapy are summarized in **Table 3**.

**Figure 6:**
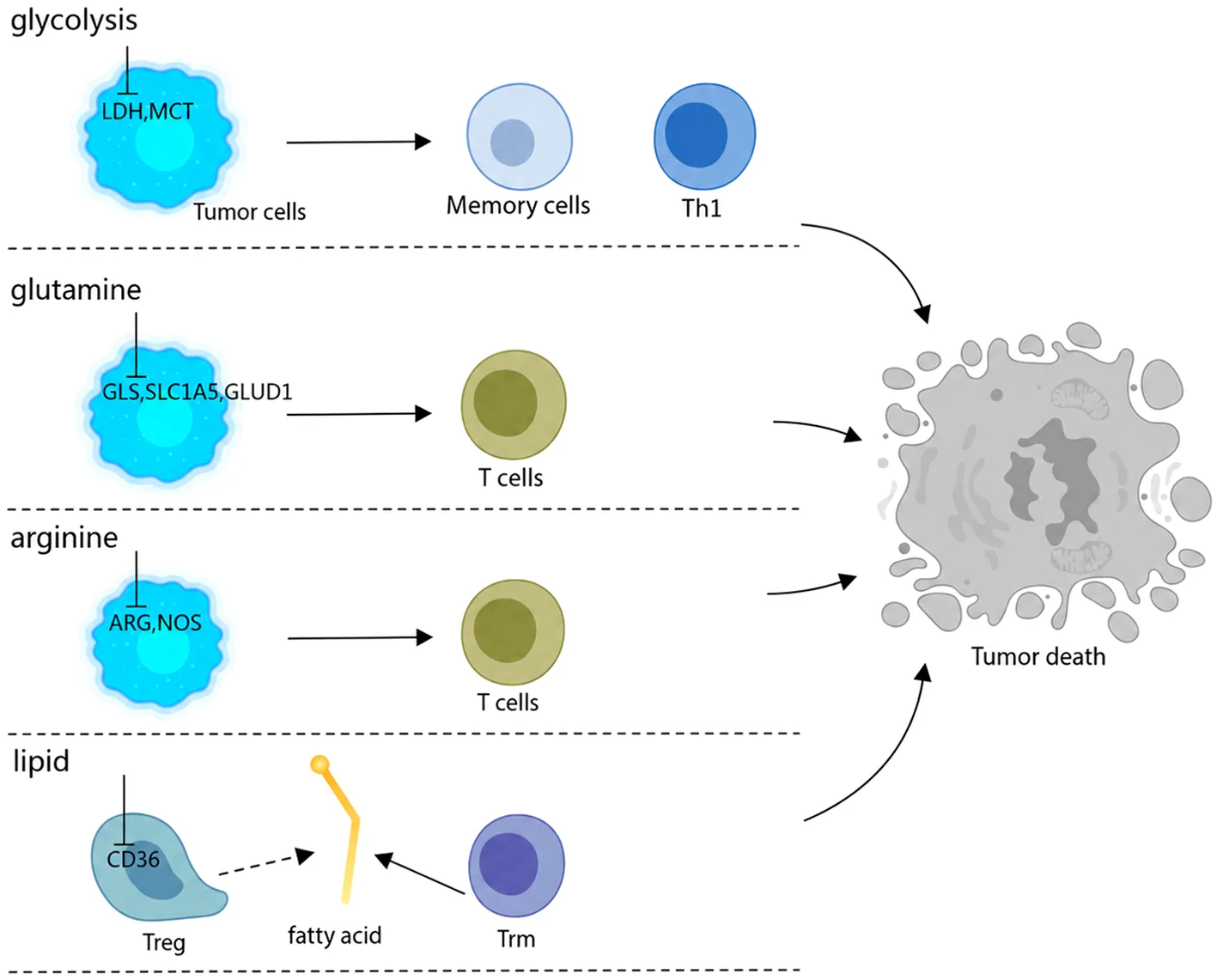
Targeting metabolic pathways in the osteosarcoma metabolic-immune microenvironment. Glycolysis/lactate transport, glutamine metabolism, arginine metabolism, and lipid homeostasis can shape immune suppression, nutrient competition, and therapeutic resistance. Rational therapeutic design should integrate metabolic remodeling with immune activation while preserving antitumor immune-cell fitness. This schematic combines OS-specific targets with representative metabolic regulators reported in broader cancer studies; the evidence levels and model-specific limitations of the corresponding pathways and interventions are discussed in Sections 4–5 and **Tables 2**, **3**.

**Table 3:** Metabolism-targeted strategies with potential to enhance immunotherapy in osteosarcoma.

Target	Representative inhibitor or strategy	Cancer type or OS evidence	Immune effect	Combination with immunotherapy	Translational status	Limitations
LDH/lactate signaling	LDH inhibition; HCAR1-PP2A modulation	OS cellular/preclinical evidence for HCAR1 signaling; broader LDH evidence is non-OS (110–114, 158, 159)	May reduce tumor lactate signaling but can also impair T-cell metabolism	No established OS ICB combination	Preclinical	Target, timing, and immune-cell toxicity remain unresolved
MCT4/lactate export	MCT4 inhibition, including VB124	Non-OS hepatocellular carcinoma evidence (115)	Reduced acidosis and increased CD8^+^ T-cell infiltration in HCC	Anti-PD-1 combination demonstrated in HCC models	Preclinical	No direct OS efficacy; transporter redundancy is possible
GLS1/glutamine use	CB-839; transport-based strategies	OS preclinical GLS1, LAT2, and SLC38A5 evidence (34, 122–124)	GLS1 inhibition targets tumor glutamine dependence, while LAT2 links macrophage signals to tumor amino-acid uptake and CD47 expression	Glutamine-ICB synergy is non-OS (81, 130)	Preclinical	Shared glutamine dependence across immune cells
Arginine axis	L-arginine supplementation; SIRPA/SLC7A3 or ARG modulation	Immunocompetent OS mouse and OS xenograft evidence (132, 133)	L-arginine supported CD8^+^ T-cell function; myeloid ARG mechanisms remain incompletely defined in OS	L-arginine combined with anti-PD-L1 showed activity in immunocompetent OS mouse models	Preclinical	No human efficacy; opposing tumor and immune requirements
FASN/SQLE	FASN or SQLE inhibition	OS cellular and preclinical evidence (145, 147)	Direct immune effects in OS are unclear	No established ICB combination	Preclinical	Normal-tissue and immune lipid requirements require evaluation
CD36/fatty-acid oxidation	CD36 blockade or fatty-acid oxidation modulation	Non-OS immune and tumor models (150, 153–157)	Effects differ across Tregs, effector, memory, and tissue-resident T cells	Potential ICB combinations are non-OS	Preclinical	Cell-type selectivity is essential
FABP5/LIPA	FABP5 interference; LIPA inhibition	OS cellular FABP5 and OS TAM LIPA evidence (35, 146)	LIPA-positive TAMs suppressed T cells in functional models	No established OS ICB combination	Preclinical	Clinical relevance and target selectivity remain unknown

Current OS studies provide preclinical support for evaluating HCAR1 and acid-adaptation pathways, GLS1 and amino-acid transporters, CSF1R and specific MDSC-associated signals, L-arginine supplementation with anti-PD-L1, and lipid-related targets such as SQLE and LIPA (33–40, 110, 122, 132, 147). MCT4 inhibition combined with anti-PD-1 and glutamine blockade combined with ICB have shown preclinical activity in non-OS models (81, 115, 130). These strategies require immune monitoring because the same metabolic pathway can support both tumor cells and antitumor lymphocytes.

Biomarker-guided selection may be more appropriate than the empirical addition of metabolic inhibitors. Potential markers include defined myeloid states, extracellular acidity, GLS1 or amino-acid transporter expression, lipid-metabolism signatures, and T-cell exhaustion. Their predictive value has not been validated prospectively in OS, and transcript abundance should not be used as a substitute for metabolic activity.

## 6 Discussion, challenges, and future perspectives

Previous OS reviews have focused on the immune microenvironment, macrophage-directed therapies, and immune checkpoints (6, 7, 160). Here, stromal and immune-cell states are considered together with glucose/lactate, glutamine, arginine, and lipid metabolism. Evidence obtained directly in OS is distinguished from mechanisms described mainly in other tumor types, while public single-cell data are used to illustrate cellular heterogeneity rather than metabolic causality.

Established OS evidence now supports several parts of this framework. Patient and model studies link glycolysis and acidosis to stromal IL-6 signaling, invasion, and metabolic adaptation (92, 93). GLS1 and amino-acid transport studies support glutamine dependence and CD47-related immune evasion (34, 122–124). Myeloid studies identify CSF1R, IL-18, SDF-1/CXCR4, IL-23, G-CSF, and S100A11-containing extracellular vesicles as experimentally defined OS pathways (33, 36–40). CAFs, ECM deposition, and selected lipid targets also have OS-specific support (19–21, 35, 145–147).

Evidence is less mature for several other components. L-arginine supplementation combined with anti-PD-L1 and SIRPA-SLC7A3 signaling have been examined in OS models, but their clinical relevance remains unknown (132, 133). Studies of T cells and NK cells in OS provide prognostic or model-specific evidence of immune activity, whereas direct links to their metabolic dependencies remain limited (73, 74, 84, 85). Similarly, MCT inhibition, CD36 targeting, fatty-acid oxidation, and glutamine-ICB combinations are supported mainly by studies in other tumor types and still require direct testing in OS.

Metabolic and immune states may also change during chemotherapy, recurrence, and metastatic progression. Accordingly, observations from treatment-naive primary tumors may not fully represent later disease states, highlighting the value of longitudinal or paired-sample analyses. Future studies should combine spatially resolved metabolite measurements with cell-state information and clinically annotated samples. Experimental models should preserve the mineralized bone niche, immune competence, and lung metastatic setting when these features are relevant. This approach may help identify patients who are more likely to benefit from specific metabolic and immune combinations, but such strategies will require direct preclinical and clinical validation before therapeutic conclusions can be drawn.

## 7 Conclusion

The metabolic-immune TME contributes to treatment resistance in metastatic, recurrent, and drug-resistant OS. Current OS-specific studies provide the clearest support for glycolysis and acidosis, glutamine dependence, defined myeloid pathways, stromal and ECM effects, and selected lipid-related mechanisms. Arginine-directed approaches, NK-cell immunometabolism, and most combinations of metabolic targeting with ICB remain preclinical or hypothesis-generating. Single-cell and spatial studies can help define testable questions, but they do not establish metabolic causality or treatment response. Further progress will depend on cell-specific validation, immune-competent OS models, metabolic biomarkers, and prospective clinical evidence.
